# Does Anticoagulant Medication Alter Fracture-Healing? A Morphological and Biomechanical Evaluation of the Possible Effects of Rivaroxaban and Enoxaparin Using a Rat Closed Fracture Model

**DOI:** 10.1371/journal.pone.0159669

**Published:** 2016-07-25

**Authors:** Peter Michael Prodinger, Rainer Burgkart, Kilian Kreutzer, Franz Liska, Hakan Pilge, Andreas Schmitt, Martina Knödler, Boris Michael Holzapfel, Alexander Hapfelmeier, Thomas Tischer, Oliver Bissinger

**Affiliations:** 1 Klinik und Poliklinik für Orthopädie und Sportorthopädie, Klinikum rechts der Isar der Technischen Universität München, Ismaninger Straße 22, D-81675, München, Germany; 2 Klinik und Poliklinik für Mund-, Kiefer- und Gesichtschirurgie, Universitätsklinikum Hamburg-Eppendorf, Martinistraße 52, 20246, Hamburg, Germany; 3 Orthopädische Klinik, Universitätsklinikum Düsseldorf, Heinrich-Heine-Universität, Moorenstraße 5, D-40225, Düsseldorf, Germany; 4 Klinik und Poliklinik für Orthopädie und Sportorthopädie, Abteilung für Sportorthopädie, Klinikum rechts der Isar der TU München, Ismaninger Straße 22, D-81675, München, Germany; 5 Regenerative Medicine Group, Institute of Health and Biomedical Innovation, Queensland University of Technology, Brisbane, Australia; 6 Institut für Medizinische Statistik und Epidemiologie, Klinikum rechts der Isar der Technischen Universität München, Ismaninger Straße 22, D-81675, München, Germany; 7 Orthopädische Klinik und Poliklinik, Universität Rostock, Doberaner Straße 142, D-18057, Rostock, Germany; 8 Klinik und Poliklinik für Mund-, Kiefer- und Gesichtschirurgie, Klinikum rechts der Isar, Technische Universität München, Ismaninger Straße 22, D-81675, München, Germany; Medical University of South Carolina, UNITED STATES

## Abstract

Low molecular weight heparin (LMWH) is routinely used to prevent thromboembolism in orthopaedic surgery, especially in the treatment of fractures or after joint-replacement. Impairment of fracture-healing due to increased bone-desorption, delayed remodelling and lower calcification caused by direct osteoclast stimulation is a well-known side effect of unfractioned heparin. However, the effect of LMWH is unclear and controversial. Recent studies strongly suggest impairment of bone-healing *in-vitro* and in animal models, characterized by a significant decrease in volume and quality of new-formed callus. Since October 2008, Rivaroxaban (Xarelto) is available for prophylactic use in elective knee- and hip-arthroplasty. Recently, some evidence has been found indicating an *in vitro* dose independent reduction of osteoblast function after Rivaroxaban treatment. In this study, the possible influence of Rivaroxaban and Enoxaparin on bone-healing *in vivo* was studied using a standardized, closed rodent fracture-model. 70 male Wistar-rats were randomized to Rivaroxaban, Enoxaparin or control groups. After pinning the right femur, a closed, transverse fracture was produced. 21 days later, the animals were sacrificed and both femora harvested. Analysis was done by biomechanical testing (three-point bending) and micro CT. Both investigated substances showed histomorphometric alterations of the newly formed callus assessed by micro CT analysis. In detail the bone (callus) volume was enhanced (sign. for Rivaroxaban) and the density reduced. The bone mineral content was enhanced accordingly (sign. for Rivaroxaban). Trabecular thickness was reduced (sign. for Rivaroxaban). Furthermore, both drugs showed significant enlarged bone (callus) surface and degree of anisotropy. In contrast, the biomechanical properties of the treated bones were equal to controls. To summarize, the morphological alterations of the fracture-callus did not result in functionally relevant deficits.

## Introduction

An immobilized patient after orthopedic surgery or trauma is an obligatory subject of anticoagulant therapy to prevent thrombosis or pulmonary embolism. Without this therapy, the rate of deep vein thrombosis following major lower extremity surgery is between 40–60%, increasing the risk of developing fatal pulmonary embolism [1, 2]. Established treatment includes heparin and low-molecular weight heparins (LMWHs), whilst Vitamin K antagonists (4-hydroxycoumarin-derivatives) are routinely used as anticoagulants to prevent thrombosis and embolism in cardiac arrhythmia or as long-term secondary prophylaxis. In 2008, Rivaroxaban was initially approved for the prevention of venous thromboembolism in adult patients undergoing elective hip and knee replacement surgery [3]. Recently, therapeutic indications were extended to the treatment of ischemic stroke, atrial fibrillation and deep venous thrombosis or pulmonary embolism increasing the clinical impact of this substance significantly [4].

In 1955, the potential effects of anticoagulants on fracture healing were studied for the first time by Stinchfield who was able to reproduce delayed unions in animals receiving heparin [5]. Thereafter, several preclinical animal studies have stated that heparin and LMWH caused decreased trabecular volume through increased resorption, a decreased rate of bone formation and lower calcification of the callus as well as delayed remodeling, presumably caused by direct osteoclast stimulation [6–11]. However, observed effects of LMWH seemed milder compared to unfractioned heparin [9, 11]. Prolonged, unfractionated heparin treatment has been associated with bone loss and an increased risk of fracture [12, 13]; while long term administration of LMWH is associated with a higher risk of developing osteoporosis [14], this is a rare adverse side effect with an incidence of 2–5% [15].

Systematic examinations on the possible effects of the direct factor Xa-inhibitor Rivaroxaban on bone healing are sparse. Solayer et al. treated primary human osteoblast cultures *in vitro* with varying concentrations of Rivaroxaban and Enoxaparin and found a significant reduction in osteoblast function independent of dose. This reduction was associated with reduced mRNA expression of osteocalcin, Runx2, and the osteogenic factor BMP-2. Though both agents did not adversely affect osteoblast viability, the authors concluded that Rivaroxaban treatment may negatively affect bone through a reduction in osteoblast function [16]. Similarly, Gigi et al. observed a dose-dependent inhibition of the DNA-synthesis and Creatine kinase-specific activity of SaOS2 cells via Rivaroxaban. Alkaline phosphatase-specific activity was decreased and cell mineralization unaffected. The *in vitro* model demonstrated a significant Rivaroxaban-induced reduction in osteoblastic cell growth and energy metabolism, indicating that Rivaroxaban might inhibit the first stage of bone formation [17].

As bone formation demands the coordination of different cell types and the activation of specific signal pathways, a single cell culture might not be sufficient to comprehend the whole process resulting in the restoration of bone. In light of the fact that heparin and LMWHs have been associated with detrimental effects on bone on the one hand and the possible inhibitory effects of Rivaroxaban, *in vitro* on the other, we aimed to investigate possible adverse effects *in vivo* via a standardized, rodent fracture model. Our main interest was the evaluation of whether Rivaroxaban or Enoxaparin alters biomechanical properties or morphological features of the newly formed callus through μCT analysis.

This study aims to investigate whether anticoagulant medication (Rivaroxaban or Enoxaparin):

1. Alters failure load, stiffness or work to failure of fracture callus in the animal model situation?
2. Causes ultrastructural changes within the callus?

## Materials and Methods

The study was performed in the animal facility of the Klinikum rechts der Isar, Technische Universität München and was evaluated and approved by the local authority (Regierung von Oberbayern, approval no. 55.2-1-54-2531-17-10) as required by German law.

70 male Wistar rats (CRL:WI), obtained from Charles River Laboratories (Sulzfeld, Germany), 15 weeks of age and weighing between 400 and 460g were used in the main experiment. After arrival, all rats were granted a 14 day acclimation period before entering the experiment. Rats were housed in open cages, polysulfone type III OTC, with a base area of 825 cm^2^ (Ehret, Emmendingen, Germany). After acclimation they were randomized and allocated to the different arms (Enoxaparin, Rivaroxaban and Controls) and groups (Group A: Biomechanical Testing, 15 animals per substance; 4 reserve. Group B: Morphometry, 7 animals per substance) of the study.

### Surgery

The rats underwent antegrade pinning of the right femur and subsequent standardized fracture. The surgical procedure performed was a modification of the method first described by Bonnarens and Einhorn in 1984 [18], but instead of opening the knee joint for retrograde pinning, a K-wire was inserted anterograde via the intertrochanteric fossa.

All animals were prepared for surgery by shaving and cleansing of the left leg. After preoperative skin-desinfection, sterile coverage of the op-site was provided. The skin incision was done over the greater trochanter. After cutting the fascia, the gluteal muscles were separated respecting the direction of fibers. The intertrochanteric fossa was palpated and the femoral neck was grasped with a curved forceps. Subsequently, a 0.6mm K-wire was drilled into the intramedullary cavity by the use of a picture amplifier. Distally, the pin reached the supracondylar region without exceeding the bone, as not to interfere with knee motion. After a terminator radiographic control in 2 planes, the pin was cut flush with the cortex and the wound closed in layers.

**Fig 1:**
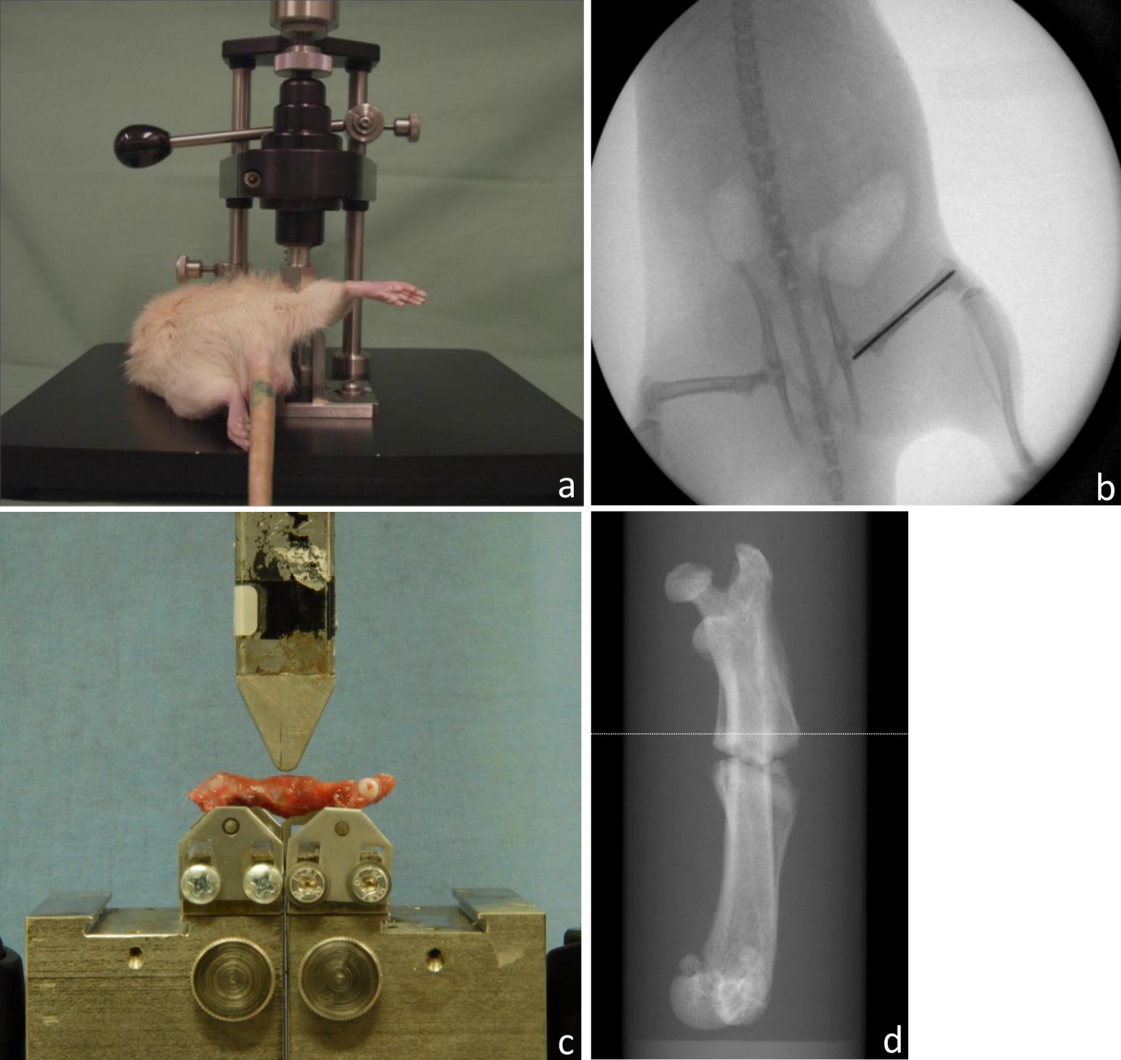
Experimental Setup. (a) Standardized fracture: Anesthetized animal placed on the fracture device. Leg placed across an open platform. (b) Postoperative radiograph, a.p. view after fracture. A transverse fracture with minimal dislocation can be seen at the middle of the femoral shaft. (c) Setting during biomechanical testing of the specimens. The distance between the bars was adapted for each bone. All bones were loaded until failure (V-max) with a persistent test velocity of 5mm/min. Meanwhile a load-displacement diagram was recorded every 0.1 second and thereby failure load was determined. (d) Scout view scan before μCT-measurement.

At the time of surgery, a standardized fracture was completed according to the method described by Bonnarens et al. [18]. A blunt guillotine 3-point bending device consisting of a 500 g weight driving the blunt guillotine down onto an outstretched rat leg placed across an open platform was used creating a 3-point bending mechanism (Fig 1a and 1b).

### Drugs

Enoxaparin was administered twice daily (every 12 hours) with 1000 IU/kg body weight, starting with the first injection 12 hours before the operation. Rivaroxaban was administered via medicated feed provided by Bayer Health Care AG, containing 600 ppm/g. The medicated fed was provided 24 hours before the operation. The chosen amount of medication and application has proven to produce comparable levels of factor Xa inhibition (pilot-studies prior to the main experiment, see supplemental data, S1 File). In detail, inhibition levels were constantly between 84% and 90% for Rivaroxaban measured over a period of 16 hours. Enoxaparin produced comparable levels of factor Xa-inhibition ranging from 74% to 80% in the first 4–5 hours after injection, efficiency was decreasing between 5 and 9 hours after injection. Therefore, it was decided to administer Enoxaparin twice daily every 12 h.

After 21 days the animals were euthanized and both femora harvested. According to group-allocation the bones were frozen and stored at -20°C (Group A, Biomechanical Testing) or fixed in 100% Methanol and stored at 4°C (Group B, Micro CT). Analyses were done after removal of the intramedullary pin, either via biomechanical testing until failure or Micro CT scan.

All researchers conducting surgery, performing x-rays, collecting samples or analyzing data were blinded throughout the duration of the entire experiment.

### Biomechanical Testing

Biomechanical examination of the specimens was done by three-point bending using a Zwick material testing machine (Zwicki 1120, Zwick GmbH & Co, Ulm, Germany). Positioning for three-point-bending was carried out with force transmission at the evaluated middle position between the distal and the proximal site of each femur (Fig 1c). Bearing- and loading-bars had a rounded tip with a diameter of 2.5mm. The distance between the bars was adapted for each bone. The femora (n = 90) were placed with their posterior surface on the lower supports of the bending apparatus. These supports were adjusted individually so that the first support was placed just distal to the trochanter minor and the other support just proximal to the condyles of the femur (popliteal plane) [19]. All bones were loaded until failure (V-max) with a persistent test velocity of 5mm/min. Meanwhile, a load-displacement diagram was recorded every 0.1 second and thereby failure load was determined. Measurement was done with a load sensor for 2.5 KN (Klasse 0.05, A.S.T. GmbH, Dresden, Germany). The stiffness was defined as linear regression with TestXpert V12 software, and deduced by obtaining the gradient of the linear part of the load-displacement curve. For the experimental sides it was defined as the interval between 2 N and 10 N, for the controls between 20 N and 80 N. Manual corrections were applied if necessary (e.g. short, linear part of the curve; later or earlier onset). Work to failure was calculated representing the area under the curve until V-max.

Failure load, stiffness and work to failure were collected for each bone of the biomechanical group. Absolute as well as relative values (failure load and stiffness of the experimental side in relation to the contralateral, healthy bone) were determined.

### Micro-CT

All analyses were done by using μ-CT 40, Scanco Medical^®^ AG, Brüttisellen, Switzerland. The samples were placed and aligned in parallel to a transparent cylindrical sample holder (18.5 mm diameter) and secured with a surrounding sponge [20]. For heat protection and preservation from drying the sample holder was filled with 100% methanol for the duration of the examination time of 61.9 minutes.

In preparation of each μ-CT analysis a scout view was used for the exact determination of the region of interest (ROI) [21] (Fig 1d). In our study the ROI covered 6.2 mm in the z-axis, 3.1mm proximal and 3.1mm distal of the fracture gap. The scans with a thickness of 0.01 mm were performed with a ‘high resolution scan mode’. The integration time was set at 200 ms. 1000 projections (each projection is sampled for 200 ms) with 2048 measurement points each were taken over 180°. The integration time corresponds to the exposition time of the detector of the x-ray of each projection is exposed to. Contouring was done manually by using a standard circle wide enough to enclose the bone of all slices. Within this ROI, thresholding was performed visually and based on histograms to separate callus, cortical bone, marrow and air. The grey-values were globally binarized using the following parameters for callus [sigma (0.8), support (1) and threshold (150)] and cortical bone [sigma (1.5), support (3) and threshold (370)] [22]. After the reconstruction of the data the analysis of the non volume-dependent parameters was performed on the basis of the selected volume of interest (VOI), to obtain the 3-dimensional interpretation (3D) [23]. All processing steps were operated automatically using Image Processing Language (IPL, Scanco Medical AG, Brüttisellen).

The following measures of bone structure and composition were evaluated from the μ-CT image data for each specimen: Bone volume (BV); tissue mineral density (TMD) = material density = bone tissue density = degree of mineralisation; bone mineral content (BMC), defined as callus BV multiplied by TMD; trabecular thickness (Tb. Th.); degree of anisotropy (DA); bone surface (BS); structure model index (SMI) [24].

### Statistics

Sample size estimation for the biomechanical testing was done according to the specifications by Leppänen et al. regarding biomechanical testing in animal models [25]. By allocating at least 14 animals per group, recognition of expected treatment effects of 10% would be feasible with statistical power of 90% at a significance level of p<0.05 in the femur shaft three-point bending test. So the group size for the biomechanical testing was defined with 15 animals per group.

Morphological examinations by Micro-CT were defined as explorative study. Therefore the minimum group size should be at least 5 animals [26].

Statistical analyses were performed with R 3.1.0 (The R Foundation for Statistical Computing, Vienna, Austria). As quantitative data showed no severe deviations from the normal distribution, descriptive statistics are given by mean ± standard deviation. Accordingly, group comparisons were performed by t-tests. The distribution of qualitative data is presented by absolute and relative frequencies and compared between groups using the χ^2^-test or Fisher’s exact test, depending on the cell counts of corresponding contingency tables. All tests were two-sided and performed in an exploratory manner on a 5% significance level.

## Results

Six rats developed a hematoma, located above the greater trochanter. Of those, 5 were in good general condition and stayed in the experiment, 1 rat had to be euthanized due to pale mucous membranes and a poor general condition. All rats developing hematoma belonged to the Enoxaparin group. So the prevalence of hematomas in the Enoxaparin group was 26% whereas no relevant hematoma could be seen in the Rivaroxaban or in the control-group (p = 0.022).

### Biomechanical Testing

**Fig 2:**
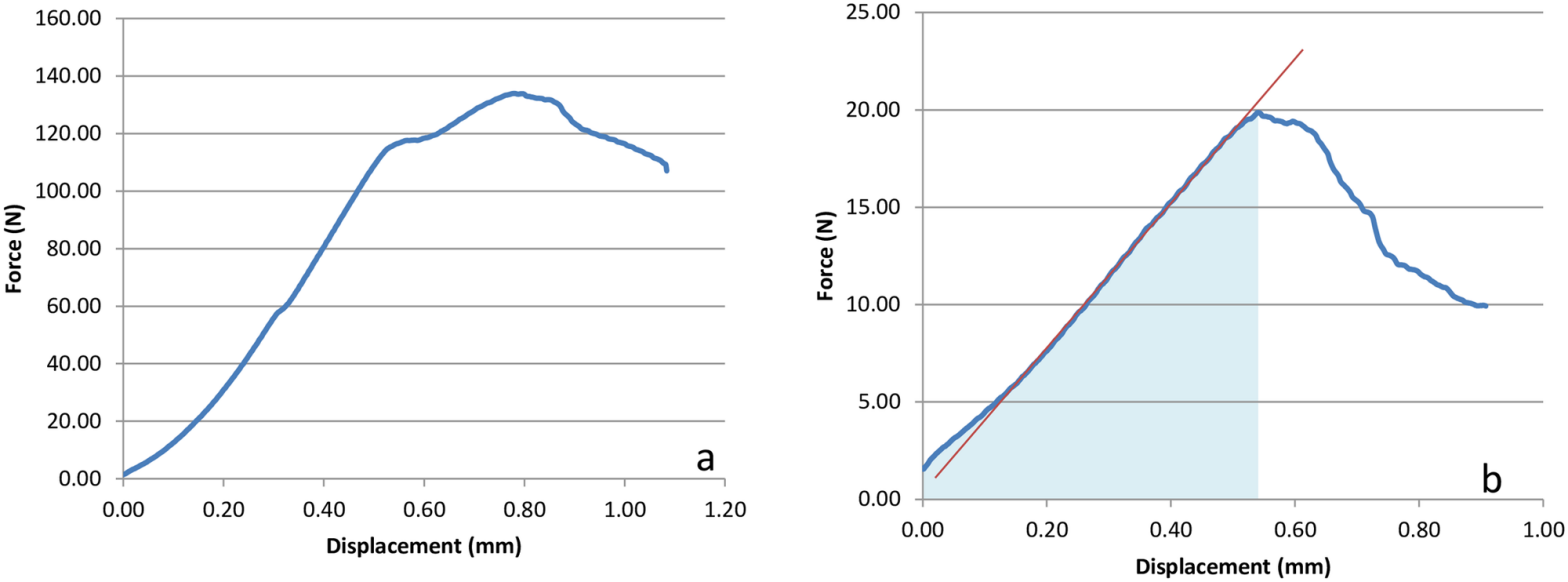
Biomechanical Parameters. (a, b) Load-displacement diagram of corresponding bones during three-point bending. The first diagram (a) shows the fracture-curve of the control side, the second the experimental side (b). The ordinate displays the force (N), the abscisse the displacement (in mm). Different scales of the ordinate. The red line in Fig 2b displays stiffness (gradient of the linear part of the load-displacement curve). The light blue area is the work to failure (area under the curve (Nmm)).

A total of 45 animals (90 femura) were investigated by biomechanical testing (Fig 2a and 2b). In all 90 femura the failure load could be determined. In 2 bones, both experimental sides (1 in the Rivaroxaban-group, 1 in the Enoxaparin-group) the stiffness could not be calculated due to absence of linear characteristics in the load-displacement diagram.

### Failure Loads (V-max)

**Fig 3:**
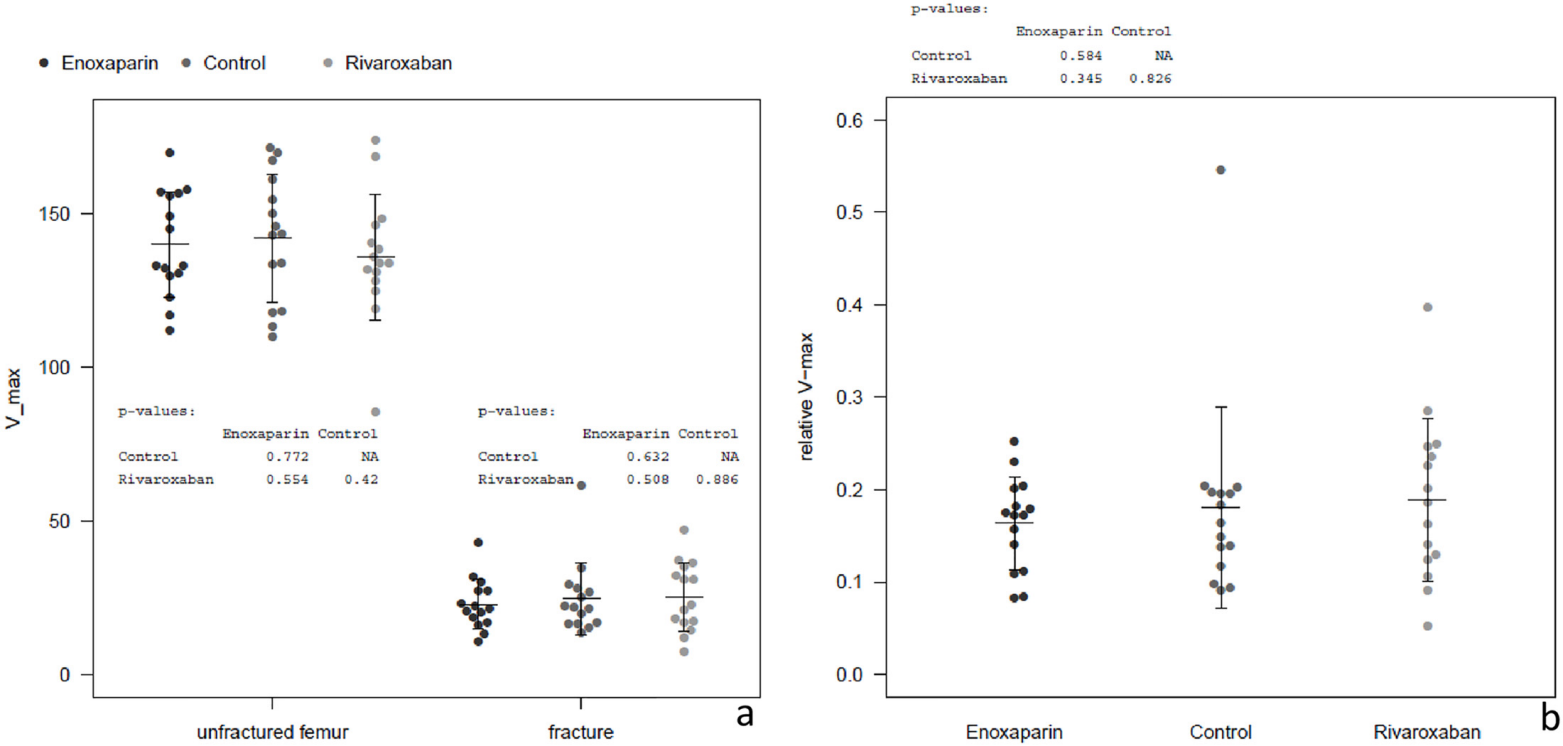
Results Biomechanical Testing, V-max absolute and ratio. (a) Dot-plots of absolute V-max values for controls, Rivaroxaban and Enoxaparin; Control sides (unfractured femur) and experimental sides (fracture). No sign. differences between controls and substances. (b) Dot-plot of ratios fractured to unfractured bones in V-max for controls, Rivaroxaban and Enoxaparin. No sign. differences between controls and substances.

Failure loads of the experimental sides averaged 24.7 ± 11.9 N for the controls, 25.3 ± 11.3 N for Rivaroxaban and 22.9 ± 8.1 N for Enoxaparin. The contralateral, unfractured bones counted up for 142.1 ± 20.8 N for the controls, 136.0 ± 20.5 N for Rivaroxaban and 140.1 ± 17.1 N for Enoxaparin (Table 1, Fig 3).

**Table 1:** Results Biomechanical Testing.

	N		unfractured femur	fracture	ratio	p-value*
V-max	15	Enoxaparin	140.1 ± 17.1	22.9 ± 08.1	0.16 ± 0.05	<0.001
(N)		Control	142.1 ± 20.8	24.7 ± 11.9	0.18 ± 0.11	<0.001
		Rivaroxaban	136.0 ± 20.5	25.3 ± 11.3	0.19 ± 0.09	<0.001
Stiffness	14	Enoxaparin	227.1 ± 29.5	18.4 ± 07.2	0.08 ± 0.03	<0.001
		Control	231.0 ± 50.5	27.8 ± 22.4	0.12 ± 0.10	<0.001
		Rivaroxaban	214.1 ± 48.1	23.1 ± 14.4	0.12 ± 0.08	<0.001
Work to failure	15	Enoxaparin	13.8 ± 4.9			
(Nmm)		Control	15.5 ± 8.3			
		Rivaroxaban	15.4 ± 6.5			

Results of biomechanical testing (Mean ± SD, *one sample / paired samples t-test). N = 15 (except for assessments involving stiffness in the Enoxaparin and Rivaroxaban groups, here n = 14). p-value displays difference between experimental and control sides.

### Stiffness

Stiffness of experimental bones averaged 27.8 ± 22.4 N/mm for controls, 23.1 ± 14.4 N/mm for Rivaroxaban and 18.4 ± 7.2 N/mm for Enoxaparin. The contralateral sides were 231.0 ± 50.5 N/mm in the control-group, 214.1 ± 48.1 N/mm in the Rivaroxaban-group and 227.1 ± 29.5 N/mm for Enoxaparin (Table 1).

### Work to Failure

**Fig 4:**
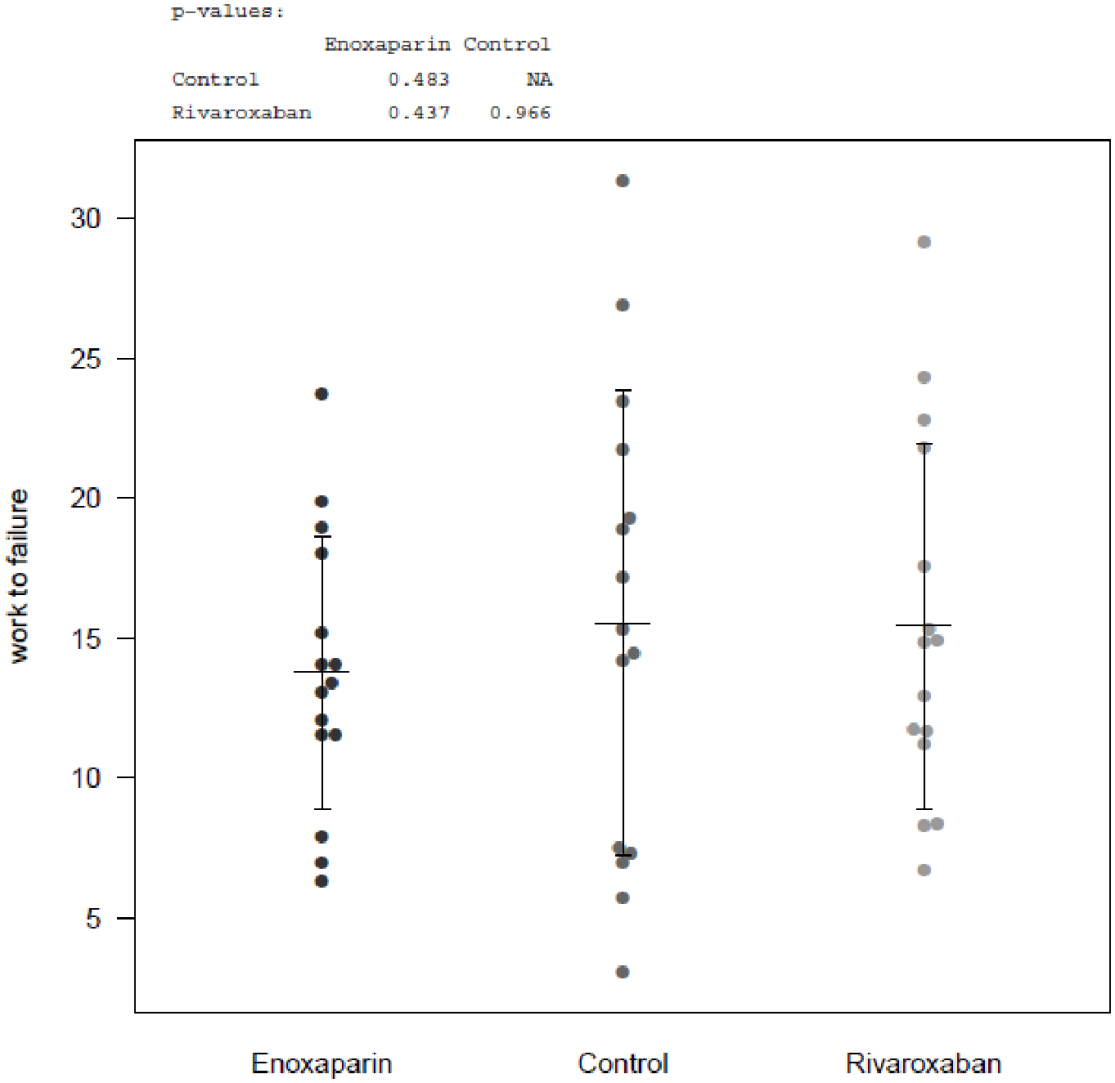
Results Biomechanical Testing, work to failure. Dot-plots of work to failure (Nmm) for controls, Rivaroxaban and Enoxaparin; experimental sides (fracture). No sign. differences between controls and substances.

Furthermore, work to failure was analyzed for experimental sides of each substance. Controls averaged with 15.54 ± 8.305 Nmm, Rivaroxaban with 15.43 ± 6.513 Nmm and Enoxaparin 13.77 ± 4.876 Nmm (Table 1, Fig 4).

### Comparison between the Groups

In general, the means and standard deviations of absolute and relative V-max, stiffness and work to failure values showed homogenous patterns throughout the groups (Table 1).

Mean V-max (failure load) of the control and experimental side ranged between 136.0–142.1 N and 22.9–25.3 N, respectively. As expected, stiffness showed a slightly higher variance in both sides and the corresponding mean values ranged from 214.1 to 231.0 and 18.4 to 27.8, respectively.

After 21 days the callus of the fractured femur reached between 16 and 19% of the strength (evaluated by obtaining V-max) of the corresponding, contralateral bone. Comparison of groups did not show significantly different mean values between the tested substances and the control (Fig 3).

### Micro-CT

**Fig 5:**
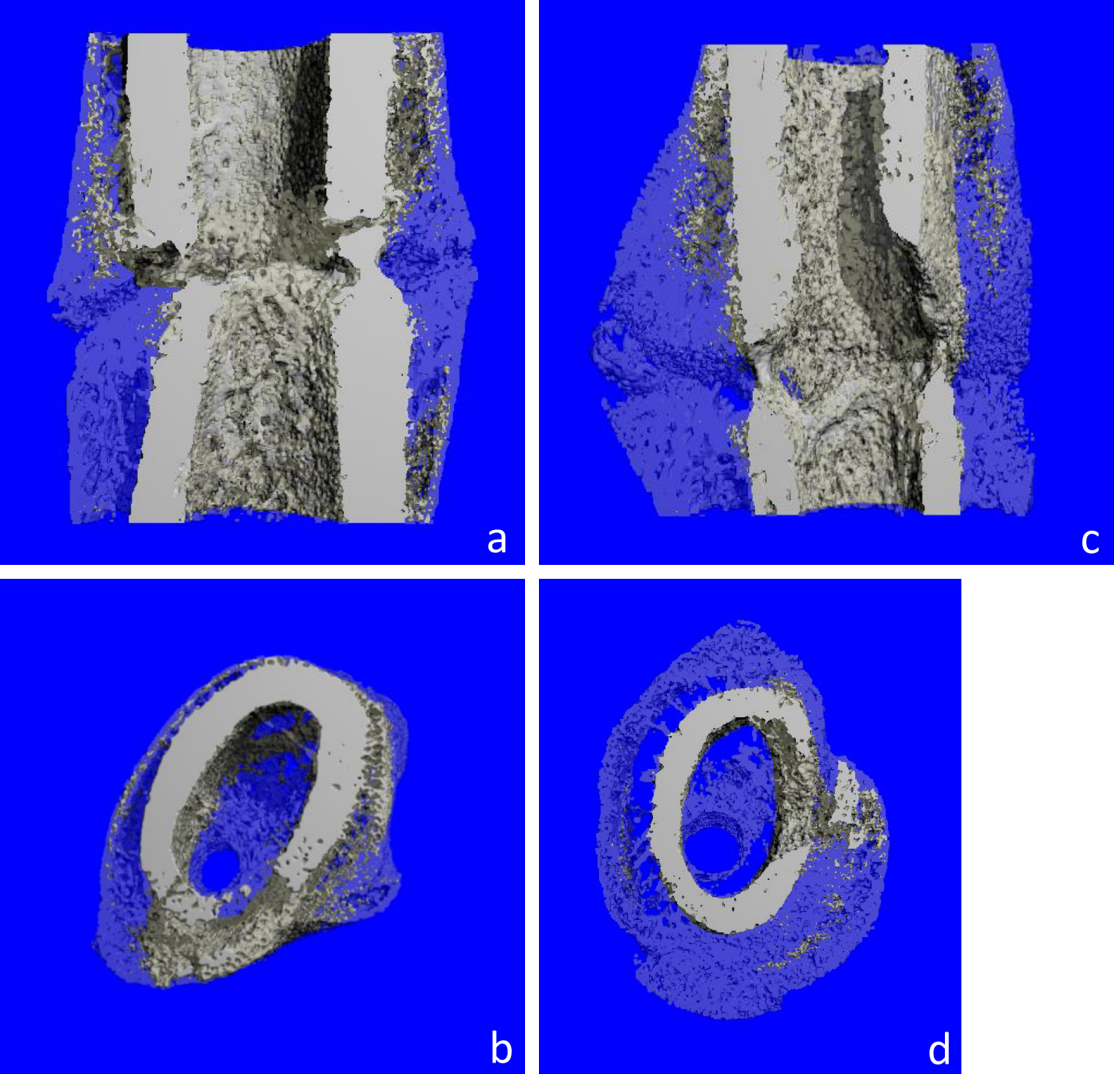
Micro-CT Scan, 3D reconstruction within the ROI. μCT scans, 3-dimensional reconstruction of ROI, virtually sliced in half or axial cuts. (a, b) representative specimen of the control group. Lower callus volume in comparison to Rivaroxaban/Enoxaparin (representative specimen, here Rivaroxaban, pictures (c) and (d)).

The results were illustrated qualitatively by three-dimensional reconstructions of representative specimens (see Fig 5) and quantitatively for each experiment. Altogether 20 bones were examined (7 in the Rivaroxaban-, 7 in the Enoxaparin-group, and 6 controls).

Absolute values of the obtained parameters are shown in Table 2.

**Table 2:** Results Micro CT Scan.

Animal/Bone	Substance	BV	Density (mg HA/ccm)	BMC	SMI	DA	BS	Tb.Th
1	Rivaroxaban	68.7	619	42.5	-0.88	1.10	1574	0.100
2		73.7	644	47.5	-0.87	1.20	1693	0.099
3		96.8	642	62.2	-0.84	1.12	2199	0.100
4		80.2	634	50.5	-1.26	1.14	1782	0.100
5		75.2	620	46.6	-1.68	1.13	1634	0.099
6		76.9	637	49.0	-1.61	1.09	1655	0.099
7		92.6	652	60.4	-1.77	1.16	1967	0.101
Mean±SD		80.6±10.3	635±12	51.2±7.3	-1.27±0.41	1.13±0.04	1786±223	0.100±0.001
8	Enoxaparin	84.8	645	54.7	-0.84	1.11	1968	0.098
9		61.1	647	39.5	-0.83	1.13	1367	0.101
10		77.3	638	49.4	-1.80	1.12	1549	0.108
11		85.9	649	55.8	-1.56	1.17	1839	0.101
12		60.8	634	38.5	-0.16	1.13	1431	0.101
13		67.2	635	42.7	-0.87	1.12	1517	0.101
14		61.9	624	38.7	-0.13	1.16	1499	0.099
Mean±SD		71.3±11.2	639±9	45.6±7.6	-0.88±0.63	1.14±0.02	1596±222	0.101±0.001
15	Control	58.2	643	37.4	-1.22	1.15	1272	0.101
16		64.3	646	41.5	-0.18	1.16	1463	0.108
17		64.1	644	40.6	-1.10	1.17	1278	0.104
18		63.9	647	42.4	-1.09	1.18	1433	0.104
19		61.3	648	39.7	-0.80	1.20	1284	0.106
20		70.7	647	45.7	-2.17	1.18	1433	0.103
Mean±SD		63.7±4.1	646±2	41.2±2.8	-1.09±0.64	1.17±0.02	1360±91	0.104±0.002

Absolute values of micro-CT parameters for each animal (bone). Displayed are bone volume (mineralized callus volume = BV), density of the callus (tissue mineral density TMD), bone mineral content BMC (defined as the callus BV multiplied by TMD), the structure model index (SMI), the degree of anisotropy DA, the bone-surface (BS) and trabecular thickness (Tb.Th).

**Fig 6:**
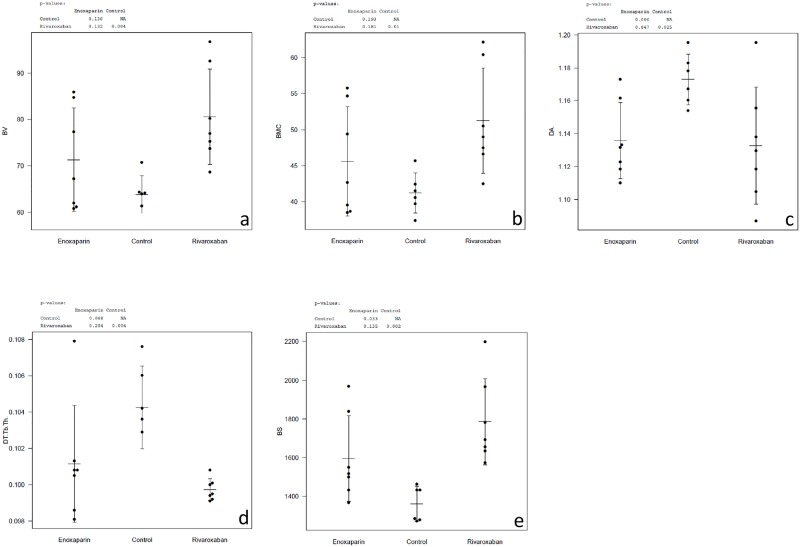
Summary Micro-CT Scan. Micro-CT based assessment of histomorphometry, black points indicate exact data, grey arbour mean data ± standard deviation. (a) Bone Volume BV (mm³) = mineralized callus volume: Significant difference of Rivaroxaban compared to control group (p = 0,004). (b) Bone Mineral Content = BMC defined as callus BV multiplied by TMD (mg hydroxyapatite/ ccm): Significant difference of Rivaroxaban compared to control group (p < 0,05). (c) Degree of Anisotropy = DA: Significant difference of both substances compared to control group (p < 0,05). (d) Trabecular Thickness = Tb-Th (mm): Significant difference of Rivaroxaban compared to control group (p < 0,05). (e) Bone Surface = BS (mm²): Significant difference of both substances compared to control group (p < 0, 05).

The quantification of bone volume (mineralized callus volume = BV) in the fractured femoral diaphysis is shown in Fig 5. The Rivaroxaban- as well as the Enoxaparin-group showed higher callus volume than the controls (sign. for Rivaroxaban, p = 0.004) (Fig 6a).

The density of the callus (tissue mineral density TMD) was lower in both groups treated either with Rivaroxaban or Enoxaparin compared to the control group. The bone mineral content BMC (defined as the callus BV multiplied by TMD) showed corresponding to BV a higher score in the treated rats. The Rivaroxaban-group showed again a statistically higher BMC than the control group (p = 0.010) (Fig 6b).

The structure model index (SMI) quantifies the plate and rod characteristic of 3D-trabecular-structure [27]. SMI is negative in case of concave surfaces, like in trabecular bone. The SMI in our investigation did not show significant changes between the groups.

The degree of anisotropy (DA) as well as the trabecular thickness (Tb.Th.) showed statistically significant lower results in the treated groups compared to the control group (Fig 6c and 6d). Bone surface (BS) is determined by triangulating the surface and calculating the total area of triangles. The surface of the bone (BS) was found significant higher in the Rivaroxaban- (p = 0.002) and the Enoxaparin-group (p = 0.033) than in the control group (Fig 6e). No statistical differences were found between Rivaroxaban and Enoxaparin for any of the callus morphometric measurements.

## Discussion

Still, there is considerable controversy concerning the question of whether anticoagulant medication, especially broadly used LMWH would potentially interfere with bone healing. In this context, Street et al. found a significant delay in fracture healing in an unstabilized rabbit rib fracture model following the administration of Enoxaparin [11]. In the Enoxaparin group, the grade of fracture healing was reduced at days 7 and 14, and the mechanical properties were weaker at day 21 compared to controls. In contrast, Hak et al. investigated Dalteparin in a standard stabilized rat femur fracture model and were not able to observe any deleterious effects [28]. Furthermore, variable effects on bone have been shown with distinct formulations of LMWHs. Fondaparinux for example was shown to cause higher mitochondrial activity and protein synthesis in osteoblasts compared to Enoxaparin and unfractionated heparin [7].

Broadly, the dose of applied medication has an impact on the results. Negative effects tend to increase with experimental overdoses. In an *in vitro* bone nodule assay, supertherapeutic doses of LMWH have been proven to decrease cancellous bone volume, demonstrated by a lack of normal remodeling and repair [29]. Supertherapeutic doses also produced a decrease in the osteoid surface area and alkaline phosphatase activity in a dose-dependent manner [9].

The mechanism of how anticoagulant medication interferes with bone-healing is still unclear. Obviously, there might be some effect on the fracture-hematoma which plays a crucial role in the restoration of fractured bones. It has been reported that routine use of LMWH in total hip arthroplasty has higher rates of surgical side hematomas [30]. Thus, the early use of LMWH in orthopedic patients suffering fractures may presumably lead to larger fracture site hematoma. As Mizuno et al. stated the osteogenic potential of the fracture side hematoma, its significance and beneficial role has been generally accepted [31]. Several studies have shown that evacuation of this hematoma can be deleterious, especially when performed several days following fracture, after the inflammatory phase has ended [32, 33]. In contrast, Street et al. observed that the high potassium (K^+^) concentration of fracture site hematoma is cytotoxic to endothelial cells and osteoblasts and therefore inhibits bone formation [34]. Only after these cytotoxic elements have undergone resorption can the angiogenic and osteogenic cytokines present within the fracture hematoma become functional. Brighton and Hunt described an area of architectural disruption and cell degradation that diminishes with the distance from the hematoma [35]. So increased fracture site hematoma volume as well as lacking hematoma may therefore provide deleterious effects on the physiological cascade of fracture healing.

Additionally, unfractioned heparin and some LMWH have been shown to provide direct, cell-mediated effects on bone formation and resorption by interfering with osteoblast- and osteoclast-function.

Unfractioned heparin has also been shown to negatively influence bone formation by promoting the activation of osteoclasts and decreasing bone volume in rats [6, 8]. This was further specified *in vitro* and could be linked to enhanced osteoclastic bone resorption through inhibition of osteoprotegerin activity [36] and induction of bone resorption in rat osteoclasts [6, 8]. Furthermore, previous studies have shown that heparin and LMWH, for example Enoxaparin, exert negative effects on alkaline phosphatase expression, as key-protein indicative for osteoblastic function [29, 37]. Ultimately, heparin seems to be able to inhibit BMP-2 osteogenic activity by binding to BMP-2 and the BMP receptor. This effect comes along with reduced Runx2, osteocalcin and alkaline phosphatase expression [38].

There is some evidence that the effects of heparin and its derivatives on osteoblast differentiation are molecular-weight dependent and increase with the molecule-mass. Handschin et al. compared the effects of Dalteparin and Fondaparinux using primary human osteoblasts. In that study, Dalteparin was found to produce a significant reduction in osteoblast differentiation and proliferation at a dose of 300μg/ml causing a decline in osteoblast specific markers, osteocalcin and alkaline phosphatase. In contrast, Fondaparinux, resembling the smaller molecule, did not produce a significant reduction in any of these variables [39]. The critical mass to affect either osteoblast differentiation or mineralization was supposed to be around 3000 Da [29, 37].

Part of the bigger impact of heparin compared to LMWH is the diverse potential to interfere with osteoclast-linages. Muir et al. for example compared the effects of the LMWH (Tinzaparin 0.5–1.0 U/g), with that of heparin (0.5 and 1.0 U/g) in Sprague—Dawley rats and found a dose-dependent decrease in cancellous bone volume for Tinazaparin and unfractioned heparin, which was dramatically higher in heparin [9]. These results were explained by the fact that while both—heparin and LMWH—decreased osteoblast number and activity, only unfractioned heparin was found to increase the number and activity of osteoclasts. The observation that heparin increases bone resorption while LMWH does not, is supported by *ex vivo* studies measuring the release of 45Ca from prelabeled fetal rat calvarias [40]. Besides, it has been shown that heparin's effects on bone are not readily reversible because it remains sequestered within the bone´s microenvironment [40].

Recently a new, direct factor Xa inhibitor (Rivaroxaban, Xarelto^®^, BAYER) is available for the prevention of venous thromboembolism in adult patients undergoing elective hip and knee replacement surgery. It has been proven to be highly effective in preventing thromboembolic events [2, 3, 41–44]. Coupled with the fact that Rivaroxaban is available orally, eliminating the need for invasive administration, Rivaroxaban is an attractive option for orthopaedic surgeons and patients alike. Regarding the efficacy of the substance, the risk of symptomatic venous thromboembolism in elective joint-replacement surgery was lowered with Rivaroxaban, but considerations of increasing the relative risk of clinically relevant bleeding with this treatment have been drawn [44], this could be relativized by pooled data in the RECORD-trials [3].

Literature concerning possible interactions of Rivaroxaban or other, direct factor Xa-inhibitors with bone-formation is sparse. Solayer et al. treated primary human osteoblast cultures *in vitro* with varying concentrations of Rivaroxaban and Enoxaparin and found a significant reduction in osteoblast function for both substances, measured by a decrease in alkaline phosphatase activity. This reduction was associated with reduced mRNA expression of the bone marker osteocalcin, the transcription factor Runx2, and the osteogenic factor BMP-2. Though both agents did not adversely affect osteoblast viability, the authors concluded that Rivaroxaban and Enoxaparin may negatively affect bone through a reduction in osteoblast function, with Rivaroxaban having the major impact [16].

Similarly Gigi et al. observed a dose-dependent inhibition of the DNA-synthesis and creatine kinase-specific activity of SaOS2 cells via Rivaroxaban. In this experiment, SaOS2 cells were treated for 24 hours with different concentrations of Rivaroxaban (0.01–50 μg/ml) and analyzed for DNA synthesis and creatine kinase- and alkaline phosphatase-specific activities. For analyzing mineralization, the treatment was extended 21 days. Rivaroxaban dose-dependently inhibited up to 60% DNA synthesis of the cells. Creatine kinase-specific activity was also inhibited dose-dependently to a similar extent by the same concentrations. Alkaline phosphatase-specific activity was dose-dependently inhibited but only up to 30%. Cell mineralization was unaffected by 10 μg/ml Rivaroxaban. The *in vitro* model demonstrated a significant Rivaroxaban-induced reduction in osteoblastic cell growth and energy metabolism and slight inhibition of the osteoblastic marker, alkaline phosphatase, while osteoblastic mineralization was unaffected. To summarize, the findings might indicate that Rivaroxaban inhibits the first stage of bone formation [17].

Contradictory, our group investigated genetical alterations by Enoxaparin and Rivaroxaban in vitro (MSC during osteogenic differentiation) and was, amongst others, able to find a significant downregulation of ALPL, Osteocalcin, BMP2, RunX2, CDH11 and SP7/OSX (osterix) for Enoxaparin but not for Rivaroxaban [45, 46].

Fusaro et al. studied the impact of warfarin and dabigatran administration on bone structure via histomorphometric analysis of unfractured vertebrae and femura in rats [47]. They were not able to show significant changes of bone-related, histomorphometric parameters of dabigatran treated rats vs. controls, Warfarin on the other hand showed substantial changes. Authors concluded that dabigatran might have a better bone safety profile than warfarin potentially resulting in a lower incidence of fractures.

In the given experiment we investigated the influence of Enoxaparin and Rivaroxaban on the biomechanical and morphological properties of new-formed callus in a standardized rodent fracture model.

The results clearly indicate two major conclusions: First, the biomechanical characteristics were not altered by the chosen, anticoagulant medication. Second, both pharmacons lead to morphological changes of the fracture-callus, precisely to an enhanced callus volume (significant for Rivaroxaban) with reduced density (trend for both substances), to a bigger bone surface (significant for both substances) and in the case of Rivaroxaban, to significantly increased bone mineral content. Furthermore, geometrical parameters such as degree of anisotropy and trabecular thickness (only Rivaroxaban significant) were different from controls. Our results are consistent with a recently published survey by Klüter et al. [48]. As Fusaro et al. [47] were not able to see histomorphometric alterations by dabigatran in unfractured bones, normal bone remodeling and fracture healing might respond differently to direct factor Xa inhibitor exposures and thus has to be evaluated differentiated.

Interpretation and conjunction of these facts seems rather complex. Several studies have used μ-CT to measure quantities such as bone volume, bone volume fraction, and mineral density in fractured callus [49–54]. However, contradictory results have been reported regarding how well these quantities predict callus mechanical properties or how these values indicate impairment of bone-healing [50, 52, 55].

Higher callus-volume has been previously associated with impaired fracture healing. Shuid et al. studied the effects of calcium supplementation on the late phase healing of fractured, osteoporotic bones using an ovariectomized rat model and were able to demonstrate higher callus volumes after eight weeks in the ovariectomized group compared to controls. Though no clear correlation to biomechanical testing (bending stress and Young’s modulus) could be found, the authors concluded that bigger callus could indicate impaired fracture healing of osteoporotic bone due to delay in callus maturation [55]. Contradictory, Morgan et al. correlated larger amounts of callus and tissue mineral density (TMD) positively with better fracture healing [24]. In their survey, the process of fracture healing was strongly linked to an increase in tissue mineral density (TMD) at every investigated time point, whereas absolute and relative amounts of mineralized tissue (BV, BMC) varied. Their results indicate that while the regain of bone strength and stiffness over time is largely due to a time-dependent increase in mineral density, this relationship between mechanical properties and mineral density can be modulated by factors that alter geometry.

TMD seems to represent one of the most important predictors of fracture healing and is strongly correlated to callus mechanical properties. Nyman et al. investigated fracture healing under anabolic treatment in a rat femur fracture model and were able to show that mineralized callus volume inversely and TMD of the callus positively correlated with peak force as determined by three-point bending, though the correlations were relatively weak [56]. The same behaviour was demonstrated in an investigation by Hao et al. [57]. The authors confirmed a significantly reduced mineralized bone volume in Micro-CT analysis in osteoporotic rat fractures, which implied a delay in fracture healing with decrease of the degree of mineralization.

Similar to studies investigating insufficient or complicated fracture healing under various circumstances [24, 55, 57], the tested types of anticoagulation, Rivaroxaban as well as Enoxaparin, showed concordant alterations of the new-formed callus, namely and in first rank to enhanced callus volume (BV) with reduced density (TMD) and to a bigger bone surface (BS). This might possibly indicate that both substances—to presume anticoagulation in general—have the potential to interfere with normal bone healing. Causally this could be linked to the hypothetically bigger fracture-hematoma under anticoagulant medication [28, 34, 35] as well as to possible, direct impairment of osteoblasts [16, 17, 29, 37]. As at least our group could not reproduce direct, osteoblast inhibition by Rivaroxaban whilst Enoxaparin did so [45, 46], we are assuming the first rather than the latter. Important questions remain open: Neither the role of the osteoclast is defined sufficiently in that context, nor is the formation or the possible alterations within the fracture hematoma. As osteoclasts play an important role in the remodeling process, further investigation should focus on them.

Approved human dosages for Enoxaparin are given between 0.6mg/kg (prophylaxis of thrombosis in elective surgery) to 2mg/kg (therapeutical dosage) bodyweight per day. This corresponds to 60–200 IU/kg per day. Extrapolations of the human dosages have been used in prior animal studies, Street et al. administered 1mg/kg Enoxaparin per day in a closed rib fracture model in rabbits and were able to prove impairment of fracture-healing [11]. Hak et al. used 70 IU/kg of Dalteparin, which represents a similar LMWH in application and indication (prophylactic dose in elective orthopedic surgery would be 5000 IU/day, conform to 70 IU/kg in humans), in a closed rat femur fracture model and observed no differences to his controls [28]. According to experimental experience, a four- to five-fold overdose of human medication is at least needed in rat models to achieve similar therapeutic results.

Rivaroxaban is a molecule of the oxazolidinone-group, showing poor hydro-solubility and a 95% to 98% plasmatic binding to albumin. Hence, determination of plasma levels is difficult and of low significance—both, in humans and in the experimental situation [58–61]. As a result, we attempted to equilibrate the two test-groups in the main experiment via adjusting a constant factor Xa-inhibition of 80%. This objective was finally achieved by injecting 1000 IU/kg bodyweight Enoxaparin twice daily on the one- and by application of Rivaroxaban feed containing 600 ppm on the other hand. In comparison to the human dosage, this means a 20-fold overdose in the experimental situation.

After 21 days the mechanical strength of the new-formed callus of the right femur reached close to 20% of the strength of the contralateral bone in 3-point bending. The significance of this result is difficult to obtain due to sparse, preexisting literature. Closest to our model comes the work of Hak et al., who tested Enoxaparin-treated femora via torsional testing after 2 and 3 weeks and obtained values between 20% and 35% of the torsional strength in accordance to the contralateral side [28]. Kaspar et al. tested two femora in a standardized rat femur-osteotomy model after two weeks, as well via torsional testing, and found percentage-torques of 9.8% and 13.8% of the contralateral bone [62]. Keeping in mind that torsional testing in general produces higher absolute values and reveals higher variance than 3-point bending, our results are well in line with prior knowledge [25, 63, 64].

With 21 days from the fracture to euthanasia, the treatment- or healing-interval of the transverse femur fracture remained relatively short. This was driven by the thought that possible alterations of the fracture-healing process might be linked with the evolution of the fracture-hematoma and should therefore predominantly interfere with the first phase of bone-healing. As resembling a transient state in the physiological process, the effect might thus be transitory and is likely to be compensated later on, especially in animals. Additionally, the *in vitro* experiment by Gigi et al. was indicative that Rivaroxaban inhibits predominantly the first stage of bone formation [17].

Having the experiment terminated after 21 days, it is obviously impossible to determine if and when measurable treatment effects adapt to untreated controls.

The applicability of our data to the human situation has certain limitations, as endochondral (via cartilage) bone formation predominates the process of fracture healing in rats whereas humans show a mixture of endochondral and intramembranous(osteoblastic) bone formation [65]. Therefore, it remains unclear whether effects in humans would be enhanced, reduced or even compensated. Nevertheless, the given experimental setup offers the possibility of studying the effects of factor Xa-Inihibitors on bone and bone-healing and could serve as experimental standard for further investigations.

As our survey focused biomechanical applications and microstructural properties of the callus, we were not able to asses changes of the cellular behavior and composition within the callus. Furthermore, examinations concerning signaling and genetic regulation within the fracture hematoma representing the first stage of bone-formation are needed to understand which cells or pathways are actually affected by anticoagulant medication.

To summarize the key-findings of our study, a bigger and somewhat disorganized callus was present in both treatment groups, which had similar biomechanical strength properties than the untreated controls. Consequently, the significance of observable morphological alterations of the callus remains unclear.

## Supporting Information

S1 File Supplemental Data—Dose-finding Studies. Summary of dose finding experiments (Pilot study 1 and 2) concerning factor Xa-inhibition of Rivaroxaban and Enoxaparin in the experimental setup.
(DOCX)

S2 File Minimal Data Sheet. Summary of biomechanical and Micro-CT test results.
(XLSX)
