# Supplementary material for: Does Anticoagulant Medication Alter Fracture-Healing? A Morphological and Biomechanical Evaluation of the Possible Effects of Rivaroxaban and Enoxaparin Using a Rat Closed Fracture Model
Source: PLoS One. 2016 Jul 25;11(7):e0159669. doi: 10.1371/journal.pone.0159669 (PMC4959754; doi:10.1371/journal.pone.0159669)
Supplement: S1 File — Summary of dose finding experiments (Pilot study 1 and 2) concerning factor Xa-inhibition of Rivaroxaban and Enoxaparin in the experimental setup. (DOCX) [file pone.0159669.s001.docx]

**S1 File: Supplemental Data - Dose-finding Studies**

**Pilot Study I:**

**Determination of plasma levels and effect of the surgical intervention**

The main objective of pilot study 1 was the determination of plasma levels and factor Xa-inhibition for both, Rivaroxaban and Enoxaparin to achieve comparable levels during the main experiment. A first dose-equivalent for Rivaroxaban was calculated on the basis of prior experience provided by Bayer Health Care AG, as well as on the basis of extrapolations of dosages used in humans. For Enoxaparin, a dose equivalent was calculated based on data provided by relevant literature [1, 2].

The second objective of pilot study 1 was determination of possible changes in Rivaroxaban plasma levels / factor Xa-inhibition in the operative setting. Especially concerning the oral administration of Rivaroxaban, altered food-intake of the animals postoperatively would provide direct impact on the main target of the study, therefore constant homogenous and comparable distribution of the pharmaca in the experimental situation had to be ensured.

For plasma level measurements of both Rivaroxaban and Enoxaparin 10 rats were used in this pilot, whereas one rat (number 10) served as a control. In order to evaluate the effects of the surgical intervention on food uptake, rats receiving Rivaroxaban were divided into two groups. Rats 1-3 did not undergo surgery. In rats 4-6 surgery was conducted in accordance with the main study and blood samples were collected during the postoperative period. Enoxaparin was administered once daily by subcutaneous injection of 350 IU/kg. Rivaroxaban was administered by medicated feed at a dosage of 1000 ppm.

**List of animals:**

**1** Rivaroxaban no surgery **4** Rivaroxaban after surgery

**2** Rivaroxaban no surgery **5** Rivaroxaban after surgery

**3** Rivaroxaban no surgery **6** Rivaroxaban after surgery

**7** Enoxaparin **10** Control

**8** Enoxaparin

**9** Enoxaparin

All rats received either the Rivaroxaban enriched fed or Enoxaparin at 10 am in the morning.

**Rats 1-3**

Rats 1, 2 and 3 were offered Rivaroxaban feed at a dosage of 1000 ppm starting at 10 am on day 1. Three blood samples were taken at an interval of 8 hours.

Rivaroxaban feed 10 am (Day 1)

1. Blood sample 6 pm (Day 1)

2. Blood sample 2 am (Day 2)

3. Blood sample 10 am (Day 2)

**Rats 4-6**

Rats 4, 5 and 6 were offered Rivaroxaban at 10 am on day 1. Surgery according to the main study was performed between noon and 2 pm the same day. For reasons of standardisation, surgical intervention was performed by one person only. Therefore, rats 4, 5 and 6 underwent surgery at different times of the day. As a consequence, whilst still at an interval of 8 hours, blood samples were taken at different times of the day as well.

**Rats 7-9**

Rats 7, 8 and 9 were administered Enoxaparin subcutaneously at a dosage of 350 IU/kg at 4 pm on day 1. The first blood sample was taken 2 hours after injection of Enoxaparin, all following blood samples were taken at an interval of 8 hours.

Enoxaparin injection 4 pm (Day 1)

1. Blood sample 6 pm (Day 1)

2. Blood sample 2 am (Day 2)

3. Blood sample 10 am (Day 2)

**Rat 10**

Rat 10 received 1 ml of sterile NaCl at 10 am. Blood samples were taken at an interval of 8 hours.

1. Blood sample 6 pm (Day 1)

2. Blood sample 2 am (Day 2)

3. Blood sample 10 am (Day 2)

Plasma levels of Rivaroxaban and Enoxaparin were than evaluated. Furthermore, activity of endogenous factor Xa was determined and a calibration curve was established using a Hyphen Assay (after a modification established by Bayer Health Care AG). Plasma levels were then converted into percentage of factor Xa-inhibition using a calibration curve. The percentage of Factor Xa-inhibition in the test samples was then evaluated using the calibration curve.

**Results**

The results of every animal at each depicted time-point are summarized in Tables 1 to 3 and Fig 1 and 2.

The analysis of the data revealed a highly different degree of factor Xa-inhibition between Enoxaparin and Rivaroxaban. Since factor Xa inhibition levels were extremely high in the Rivaroxaban group, specimens had to be diluted 1:10 and the measurements were repeated with the diluted samples. 4 hours after administration, factor Xa-inhibition measured between 44 and 62 % for Rivaroxaban (diluted samples, 1:10), whereas in rat number 4 levels were as low as 11 %. We link this to the fact that this particular animal showed a poor general condition postoperatively that might have been the consequence of a long and complicative surgical intervention causing considerable blood loss. In comparison, 350 IU/kg of Enoxaparin only resulted in 8-13 % of factor Xa-inhibition (diluted samples, 1:10).

**Table 1. Results of Factor Xa-inhibition, Rivaroxaban, diluted samples.**

| **Rat Nr.** | **Substance** | **OP** | **Dilution of plasma** | **% FXa-Inhibiton** |
| --- | --- | --- | --- | --- |
| **1/1** | **Rivaroxaban** | **pre** | **1:10** | **47** |
| **1/2** |  |  |  | **57** |
| **1/3** |  |  |  | **46** |
| **2/1** | **Rivaroxaban** | **pre** | **1:10** | **44** |
| **2/2** |  |  |  | **51** |
| **2/3** |  |  |  | **54** |
| **3/1** | **Rivaroxaban** | **pre** | **1:10** | **45** |
| **3/2** |  |  |  | **48** |
| **3/3** |  |  |  | **41** |
| **4/1** | **Rivaroxaban** | **post** | **1:10** | **11** |
| **4/2** |  |  |  | **6** |
| **4/3** |  |  |  | **1** |
| **5/1** | **Rivaroxaban** | **post** | **1:10** | **62** |
| **5/2** |  |  |  | **49** |
| **5/3** |  |  |  | **43** |
| **6/1** | **Rivaroxaban** | **post** | **1:10** | **62** |
| **6/2** |  |  |  | **27** |
| **6/3** |  |  |  | **35** |

Table 1: Results of factor Xa-inhibition of animals 1 to 6 (Rivaroxaban) at different time points in the pre- and postoperative phase.

**Fig 1. % of Factor Xa Inhibition, Rivaroxaban (Rat 1-6), dilution 1:10.**

Fig 1: Results of factor Xa-inhibition of animals 1 to 6 (Rivaroxaban) at the three different time points in the pre- and postoperative phase (Rat Nr. / time-point).

**Table 2. Results of Factor Xa-inhibition, Enoxaparin, diluted samples.**

| **Rat Nr.** | **Substance** | **OP** | **Dilution of plasma** | **% FXa-Inhibiton** |
| --- | --- | --- | --- | --- |
| **7/1** | **Enoxaparin** | **none** | **1:10** | **8** |
| **7/2** |  |  |  | **-1** |
| **7/3** |  |  |  | **2** |
| **8/1** | **Enoxaparin** | **none** | **1:10** | **13** |
| **8/2** |  |  |  | **2** |
| **8/3** |  |  |  | **1** |
| **9/1** | **Enoxaparin** | **none** | **1:10** | **9** |
| **9/2** |  |  |  | **-3** |
| **9/3** |  |  |  | **-2** |

Table 2: Results of factor Xa-inhibition of animals 7 to 9 (Enoxaparin) at different time points (no surgical intervention, dilution of plasma).

**Table 3. Results of Factor Xa-inhibition, Enoxaparin, undiluted samples.**

| **Rat Nr.** | **Substance** | **OP** | **Dilution of plasma** | **% FXa Inhibition** |
| --- | --- | --- | --- | --- |
| **7/1** | **Enoxaparin** | **none** | **none** | **66** |
| **7/2** |  |  | **none** | **16** |
| **7/3** |  |  | **none** | **11** |
| **8/1** | **Enoxaparin** | **none** | **none** | **68** |
| **8/2** |  |  | **none** | **11** |
| **8/3** |  |  | **none** | **-6** |
| **9/1** | **Enoxaparin** | **none** | **none** | **72** |
| **9/2** |  |  | **none** | **13** |
| **9/3** |  |  | **none** | **13** |

Table 3: Results of factor Xa-Inhibition of animals 7 to 9 (Enoxaparin) at different time points (no surgical intervention, no dilution of plasma).

**Fig 2. % of Factor Xa-inhibition, Enoxaparin (Rat 7-9).**

Fig 2: Results of factor Xa-inhibition of animals 7 to 9 (Enoxaparin) at different time points (no surgical intervention, Rat Nr. / time-point). Blue bars undiluted plasma, red bars dilution 1:10.

Interpretation of data lead to following conclusions:

First, the food uptake postoperatively under normal, postoperative circumstances with the animal in good shape and condition was predictable and therefore sufficient factor Xa-inhibition could be guaranteed for the main experiment.

Second, estimated doses for adjusting factor Xa-inihbition in both groups revealed different values, being excessively high in the Rivaroxaban group and declining to fast in the Enoxaparin group. Since the originally estimated dosages showed to be insufficient, a second pilot study was planned to adjust dosages for Enoxaparin and Rivaroxaban.

**Pilot Study II**

**Adjusting comparable Factor Xa-inhibition levels**

As the originally estimated dosages used in Pilot Study I showed to be not suitable, dosages for both substances needed to be readjusted in order to achieve a comparable level of factor Xa-inhibition. Therefore we planned to assess factor Xa-inhibition using different amounts of Enoxaparin measured at different time-points in comparison to rats receiving Rivaroxaban feed containing 600 ppm of the substance.

In this Pilot, a total number of 11 rats were used; whereas 6 rats (rats 1-6) were administered Enoxaparin and 5 rats (rats 7-11) were fed Rivaroxaban at the dosages shown below.

**List of animals:**

**1** 350 IU/kg subcutaneously **7** 600 ppm Rivaroxaban

**2** 350 IU/kg subcutaneously **8** 600 ppm Rivaroxaban

**3**  500 IU/kg subcutaneously **9** 600 ppm Rivaroxaban

**4**  500 IU/kg subcutaneously **10** 600 ppm Rivaroxaban

**5** 1000 IU/kg subcutaneously **11** 600 ppm Rivaroxaban

**6** 1000 IU/kg subcutaneously

With Enoxaparin being administered subcutaneously, it was necessary to monitor pharmacokinetic properties closely to adjust dosage and frequency of applications. This was achieved by frequent measurements over time as shown below (Fig 3 and 4).

**Fig 3. Illustration of blood sampling over time for Rivaroxaban.**

**Rivaroxaban-Feed**

**0h_________________12h__________________16h_______________20h_________________24h________________28h**

**↓ ↓ ↓ ↓ ↓**

**1.BS 2.BS 3.BS 4.BS 5.BS**

BS = blood sample

1. BS 2 pm local time

2. BS 6 pm local time

3. BS 10 pm local time

4. BS 2 am local time

5. BS 6 am local time

Fig 3: Blood samples were collected from rats 7 – 11 as outlined above. The first blood sample was collected 12 h after administration of Rivaroxaban-feed. All subsequent blood samples were taken at intervals of 4 h.

**Fig 4. Illustration of blood sampling over time for Enoxaparin.**

**2. Application Enoxapari**

**1. Application Enoxaparine Enoxaparin**

**0h__________1h________________3h________________5h________________9h________________12h_________15h**

**↓ ↓ ↓ ↓ ↓ ↓**

**1.BS 2.BS 3.BS 4.BS 5.BS 6.BS**

BS = blood sample

Fig 4: Blood was drawn from rats 1-6 as outlined above. The first administration of Enoxaparin was performed at 0h. The first blood sample (BD) was collected 1h after initial administration of Enoxaparin, with following blood samples at time points 3h, 5h and 9h. The 5^th^ blood sample was collected immediately prior to the second application of Enoxaparin at 12h for determination of through levels.

**Results**

Levels of factor Xa-inhibition were evaluated using the calibration curve that was established during Pilot Study I and are displayed in Tables 4 and 5 and Fig 5 and 6.

**Table 4. Results of Factor Xa-inhibition, pilot study 2, Enoxaparin, undiluted samples.**

|  | Time after injection (h) / FXa-Inhibition (%) mean | | | | | |
| --- | --- | --- | --- | --- | --- | --- |
| Enoxaparin (no dilution of plasma) | 1 h | 3 h | 5 h | 9 h | 12 h | 15 h |
| 350 IU/kg | 53 | 55 | 39 | 17 | -1 | 53 |
| 500 IU/kg | 60 | 68 | 58 | 24 | 8 | 59 |
| 1000 IU/kg | 76 | 80 | 74 | 35 | 5 | 76 |

Table 4: Inhibition of factor Xa with different amounts of Enoxaparin measured at different time-points.

**Table 5. Results of Factor Xa-inhibition, pilot study 2, Rivaroxaban.**

|  | Time (h) / FXa Inhibition (%) mean | | | | |
| --- | --- | --- | --- | --- | --- |
| Rivaroxaban (600 ppm) | 12 h | 16 h | 20 h | 24 h | 28 h |
| No dilution of plasma | 84 | 86 | 89 | 90 | 85 |
| SD (1:10) | 7 | 5 | 3 | 1 | 3 |

Table 5: Inhibition of factor Xa with 600 ppm Rivaroxaban measured at different time-points.

**Fig 5. Results of Factor Xa inhibition, pilot study 2, Enoxaparin (rats 1-6), undiluted samples.**

Fig 5: Inhibition of factor Xa with different amounts of Enoxaparin measured at different time-points. The first blood sample (1) was collected 1h after initial administration of Enoxaparin, with following blood samples at time points 3h, 5h and 9h. The 5^th^ blood sample was collected immediately prior to the second application of Enoxaparin at 12h for determination of through levels.

**Fig 6: Results of Factor Xa inhibition, pilot study 2, Rivaroxaban (rats 7-11).**

Fig 6: Inhibition of factor Xa with 600 ppm Rivaroxaban measured at different time-points (12, 16, 20, 24 and 28 hours after begin of application). Pink bars are undiluted samples; hatched bars reveal dilution of 1:5.

The analysis of the data revealed that 600 ppm of Rivaroxaban lead to a constant level of factor Xa-inhibition that was comparable to the level produced by 1000 IU/kg Enoxaparin. Inhibition levels were between 84% and 90 % for Rivaroxaban measured over a period of 16 hours. Although Enoxaparin produced comparable levels of factor Xa-inhibition ranging from 74% to 80 % in the first 4-5 hours after injection, efficiency was decreasing between 5 and 9 hours after injection. 12 hours after injection of Enoxaparin, inhibition of Factor Xa was not measurable. Therefore, it was agreed upon, to administer Enoxaparin twice daily every 12 h in the main study.

**References**

1. Shaughnessy SG, Hirsh J, Bhandari M, Muir JM, Young E, Weitz JI. A histomorphometric evaluation of heparin-induced bone loss after discontinuation of heparin treatment in rats. Blood. 1999;93(4):1231-6. Epub 1999/02/09. PubMed PMID: 9949165.

2. Street JT, McGrath M, O'Regan K, Wakai A, McGuinness A, Redmond HP. Thromboprophylaxis using a low molecular weight heparin delays fracture repair. Clin Orthop Relat Res. 2000;(381):278-89. Epub 2000/12/29. PubMed PMID: 11127666.
